# Assessment of antimicrobial use and stewardship practices among animal health practitioners, veterinary drug retailers and cattle keepers in Mvomero, Tanzania

**DOI:** 10.3389/frabi.2025.1688828

**Published:** 2026-01-23

**Authors:** Asimwe M. Mugyabuso, Isaac Makundi, Abubakar S. Hoza

**Affiliations:** Department of Veterinary Microbiology, Parasitology & Biotechnology, Sokoine University of Agriculture, Morogoro, Tanzania

**Keywords:** antimicrobial resistance, knowledge, practices, attitude, antimicrobial stewardship

## Abstract

**Background:**

Antimicrobial misuse in livestock is a key driver of antimicrobial residues and resistance (AMR), yet knowledge, attitudes, and practices (KAP) among cattle keepers and stewardship awareness among animal health practitioners (AHPs) and veterinary drug retailers ((VDR) remain poorly characterized in many low-resource settings.

**Methods:**

A total of 322 participants were interviewed in a cross-sectional study using semi-structured questionnaires and open-ended interviews. They included, 299 cattle keepers, 10 AHPs and 13 VDR. Descriptive statistics were done to compute frequencies of responses, chi square tests and linear regression analysis to assess association between dependent and independent variables while thematic analysis to analyze key informants’ interviews.

**Results:**

Awareness of antimicrobial use (AMU), residues, and AMR was generally low, with a mean score of 94 (31.4%, 95%CI: 26.2-36.6). The Mean awareness score from Linear regression showed that higher education (secondary: β = 0.878, p = 0.002; tertiary: β = 1.469, p < 0.001) and longer livestock experience (>4 years: β = 1.35, p < 0.001) were positively associated with awareness, whereas younger age groups had lower scores. Awareness significantly predicted attitudes toward responsible AMU, particularly regarding residues (β = 6.427, p < 0.001) and AMR (β = 2.473, p < 0.001). Attitudes were generally low, with an overall mean score of 2.06 (41.2%). Male sex, older age, higher education, and longer livestock experience were positively associated with more favorable attitudes. Practices were suboptimal: 99.7% (95%CI: 99.1-100) reported AMU, but only 21.1% (95%CI: 16.4-25.8) kept treatment records, hygiene was limited, and manure was frequently applied to fields (77.6%, 95%CI: 72.8-82.3). Key informants highlighted frequent non-prescription sales (AHPs: 9/10; VDR: 13/13), reliance on empirical diagnosis (AHPs: 10/10; VDR: 3/10), weak regulatory enforcement (AHPs: 8/10; VDR: 11/13), and limited knowledge of AMR (AHPs: 6/10; VDR: 3/13) as major drivers of inappropriate AMU.

**Conclusion:**

Awareness, age, education, and livestock experience significantly influenced attitudes toward responsible AMU. Systemic gaps in veterinary services emphasize the need for integrated educational and regulatory interventions to improve antimicrobial stewardship and mitigate AMR risks.

## Background

Antimicrobial agents are widely used in livestock production to treat, prevent, and control infectious diseases (Nonga et al., 2010). While these drugs are essential for maintaining animal health and productivity, their misuse can lead to the emergence of antimicrobial resistance (AMR), which poses a threat to both animal and human health (Food and Agriculture Organization of the Nations (FAO) and World Health Organization (OMS), 2018; Coque et al., 2023). AMR occurs when microorganisms develop the ability to survive exposure to antimicrobial agents, reducing the effectiveness of treatments and increasing the risk of disease spread (Mpina and Mgonja, 2024). In livestock systems, improper dosing, inadequate adherence to withdrawal periods, and empirical treatment practices contribute to the accumulation of antimicrobial residues in animal products, further amplifying public health risks (Frida et al., 2017; Azabo et al., 2022). Globally, AMR contributes to nearly five million deaths annually, with the greatest burden observed in sub-Saharan Africa (Caudell et al., 2022). Antimicrobial stewardship (AMS) refers to coordinated interventions designed to improve and measure the appropriate use of antimicrobials by promoting optimal drug selection, dosing, treatment duration, and administration routes (Mangesho et al., 2021). However, veterinary services in low- and middle-income countries often face challenges, including limited diagnostic capacity, empirical treatment practices, non-prescription sales, weak regulatory enforcement, poor record-keeping, unsafe drug disposal, poor hygiene, and client pressure, all of which contribute to inappropriate use of antimicrobials (Caudell et al., 2022; Jaime et al., 2022; Kisoo et al., 2023). Global organizations, including WHO, FAO, WOAH and UNEP advocate for AMS using a One Health approach, recognizing that AMR arises from interconnected human, animal, and environmental health systems (WHO, W. H. O. et al., 2019).

Tanzania has taken steps to address AMR through its National Action Plans on AMR (NAP-AMR), with the first phase from 2017 to 2022 and the current phase from 2023 to 2028 (Health, M. of;, & Livestock and Fisheries, M. of., 2022). The plans aim to increase awareness, strengthen surveillance, promote responsible antimicrobial use, and ensure sustainable investment in AMR control measures (Frida et al., 2017). Despite these efforts, AMS implementation in the livestock sector remains limited due to weak regulatory oversight, restricted diagnostic support, and easy access to antimicrobials over the counter (Mdegela et al., 2021; Azabo et al., 2022). As a result, livestock keepers often rely on untrained personnel, self-administer treatments, or purchase drugs from unregulated outlets, which increase the risk of residues and resistance (Pinto Ferreira et al., 2022; Akhila et al., 2025).

Mvomero District is an important livestock producing area with a large population of pastoralist communities, including the Maasai, who rely heavily on cattle for their livelihoods (URT, 2017). The high livestock density consisting approximately 207,410 cattle increases the demand for antimicrobials and can inadvertently encourage inappropriate use, particularly where veterinary oversight is limited and knowledge about residues and AMR is low (Kimera et al., 2020; Jaime et al., 2022). Although studies in other regions of Tanzania have explored knowledge, attitudes, and practices (KAP) related to antimicrobial use, there is limited evidence for Mvomero District. Understanding KAP among cattle keepers, veterinary drug retailers, and animal health practitioners is important for addressing antimicrobial misuse and mitigating the development of AMR in livestock systems (Gilbert et al., 2021; Kemp et al., 2021).

This study was intended to assess knowledge, attitudes, and practices on antimicrobial use among cattle keepers, as well as stewardship awareness and adherence among veterinary drug retailers and animal health practitioners in Mvomero District, Tanzania. The findings are intended to guide interventions that encourage responsible antimicrobial use and support broader efforts to reduce AMR in the livestock sector.

## Materials and methods

### Study area

**Figure 1:**
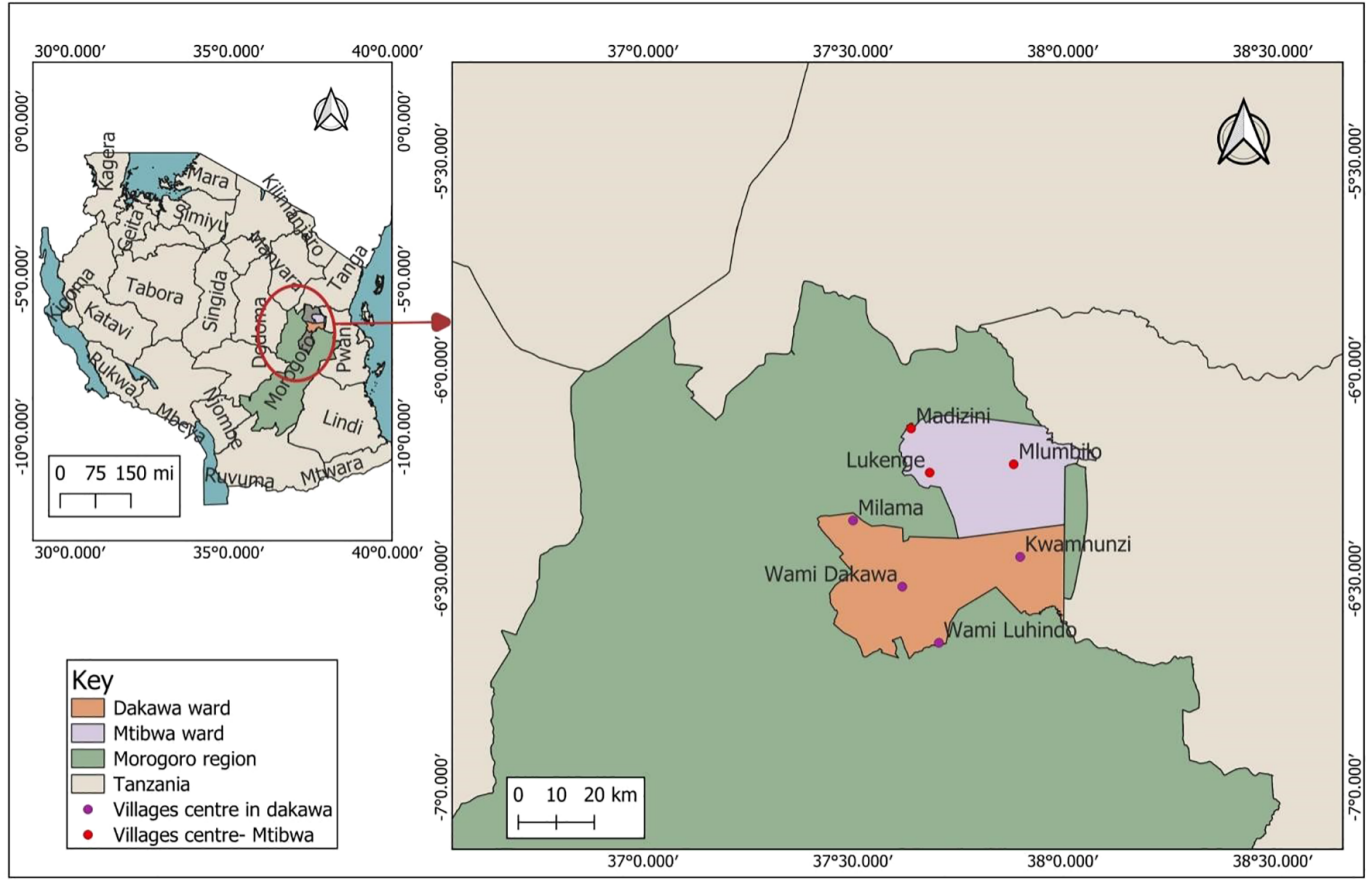
Map showing selected villages in Dakawa and Mtibwa wards, Mvomero District, Morogoro Region, Tanzania.

This study was conducted in Mvomero district, located in the Morogoro region of Tanzania. Geographically, the district lies between latitudes 05°45’ S and 07°26’ S, and longitudes 37°09’ E and 38°07’ E, covering an area of 6,631 square kilometers. Mvomero is known for its diverse agricultural landscapes and livestock farming communities. It is a home to smallholder livestock keepers, including Maasai pastoralists who depend on livestock for their livelihoods. Dakawa and Mtibwa wards were selected for this study due to their large populations of livestock keepers and the presence of livestock markets in Mtongani, Melela, and Misongeni, where substandard drugs are reportedly sold (URT, 2017). The selected villages from the two wards are shown in **Figure 1**.

### Study design and sample size determination

A cross-sectional study was conducted between February and May 2025, employing both qualitative and quantitative methods. A semi-structured questionnaire was used to assess knowledge, attitudes, and practices on antimicrobial use, antimicrobial residues, and antimicrobial resistance among cattle keepers. Only respondents aged 18 years and above who gave informed verbal consent to participate were included in the study.

The sample size for cattle keepers was calculated using Cochran’s formula for estimating proportions in an unknown population:

$n\frac{Z^{2}p(1-p)}{d^{2}}$ (Mpina and Mgonja, 2024). Where *n* is the required sample size, *Z* is the standard normal deviate (1.96 for a 95% confidence level), *p* is the expected knowledge level (assumed to be 0.5), and *d* is the desired precision (0.05).

The minimum sample size was calculated as 296 cattle keepers to achieve 95% confidence with 5% precision (Mpina and Mgonja, 2024). An additional three more respondents volunteered to participate, and the final sample size was 299.

On the other hand, Key informant interviews were conducted with 10 animal health practitioners and 13 veterinary drug sellers to explore antimicrobial use (AMU) practices and stewardship challenges.

### Sampling methodology

Records of cattle keepers were obtained from the Mvomero District Agriculture, Livestock, and Fisheries Office. A purposive sampling was used to select the villages of interest for the study. Individual cattle farmers were selected randomly in each village. Also, purposive sampling was applied for selecting veterinary drug retailers and animal health practitioners, which included both veterinary paraprofessionals and non-veterinary paraprofessionals. Due to the limited number of individuals in these two key informants’ groups within the study area, all available and consented participants were included.

### Operational definitions

To ensure clarity and consistency in data collection and analysis, key terms used in this study were operationally defined. Awareness referred to the recognition of antimicrobials and the concept of antimicrobial resistance without detailed understanding of correct use, dosage, withdrawal periods, or preventive measures. Knowledge was defined as understanding of proper antimicrobial use, including dosage, treatment duration, withdrawal periods, potential residues, and implications for antimicrobial resistance. Attitude encompassed participants’ perceptions and beliefs regarding antimicrobial use, including their willingness to follow recommended practices and seek veterinary guidance. Practice referred to actual behaviors in antimicrobial administration, record-keeping, and adherence to guidelines. Empirical treatment described the use of antimicrobials based on clinical signs alone without laboratory confirmation, and antimicrobial stewardship (AMS) encompassed coordinated actions to optimize antimicrobial use, including diagnosis, proper prescription, adherence to treatment protocols, record-keeping, hygiene, and professional education.

### Data collection tool and validation

Both the semi-structured questionnaire and the key informant’s interview guides were pre-tested in a non-selected village of the study area in Dakawa (Wami Sokoine), to validate the data collection tools. Following the pilot study, slight modifications were made to improve question clarity and ensure the instruments effectively captured all relevant information before being rolled out to the final study population.

Data collection was conducted by the researcher with the assistance of one experienced field assistant who had a background in animal Health and Production education. The questionnaire for cattle keepers consisted of 47 questions, which covered Socio-demographic information (gender, age, education, and marital status), animal husbandry, management practices, and KAP related to antimicrobial use. For key informant interviews, AHPs were asked 10 open-ended questions; veterinary drug retailers were asked 15 open-ended questions.

### Data management and analysis

Questionnaire data were managed in Microsoft Excel before importing into EpiInfo 7.2.6.0 for descriptive and inferential statistics. Descriptive statistics were used to assess the frequency of responses for each variable. The scores for Knowledge were determined by summing responses for each question. The correct answer was scored 1, while the incorrect answer was assigned a zero (0) score. Attitudes toward food safety were measured using a five-point Likert scale (1 = Strongly Disagree to 5 = Strongly Agree). Negatively phrased items were reverse-coded, and descriptive statistics (frequencies, proportions, and mean scores) were generated. A composite measure of positive attitude was calculated by combining Agree/Strongly Agree responses for positive items and Disagree/Strongly Disagree for negative items. Interpretation of mean scores followed KenPro (2020): 1.0-2.4 (negative), 2.5-3.4 (neutral), and 3.5-5.0 (positive). Associations between attitudes and sociodemographic characteristics (sex, age, education, experience, location) were examined using chi-square tests and linear regression. Key informant interviews were conducted with 10 animal health practitioners (AHPs) and 13 veterinary drug sellers (VDR) to explore antimicrobial use (AMU) practices and stewardship challenges. The data were analyzed using a semantic, inductive approach to thematic analysis following Braun and Clarke (2006). Transcripts were read multiple times to ensure familiarity, and line-by-line coding was conducted. Codes were alliteratively grouped into overarching themes and sub-themes, with frequencies of endorsement noted to indicate prevalence.

## Results

### Socio-demographic information of cattle keepers

The study involved 299 cattle keepers from Mtibwa and Dakawa wards, respectively as presented in (**Table 1**). Our results show that Wami Dakawa and Lukenge villages had the highest number of cattle keepers, contributing to 21.7% (95%CI: 17.0-26.5) and 24.7% (95%CI: 119.1-29.5), respectively. The socio-demographic profiles of cattle keepers are shown in **Table 1**. The majority of cattle keepers were male (91.3%, 95%CI: 88.3-94.3). Also, (47.5%, 95%CI: 41.9-53.1) of the cattle keepers had primary education. Relatively, more participants had more than four years of livestock-keeping experience.

**Table 1:** Socio-demographic characteristics of cattle keepers in Mtibwa and Dakawa wards, Mvomero District (N = 299).

Variable	Category	Frequency (n)	%	95%CI
Ward				
Mtibwa	Lukenge	74	24.7	19.9 – 29.5
	Mlumbilo	32	10.7	7.2 – 14.2
	Madizini	11	3.7	1.6 – 5.9
	Wami Dakawa	65	21.7	17.0 – 26.5
Dakawa	Kwa mhunzi	60	20.1	15.5 – 24.7
	Wami luhindo	35	11.7	8.0 – 15.4
	Milama	22	7.4	4.4 – 10.4
Age	18-35	52	17.4	13.1 – 21.8
	36-45	98	32.8	27.6 – 38.0
	46-55	101	33.8	28.6 – 39.1
	56-64	44	14.7	10.8 – 18.6
	≥65	4	1.34	0.0 – 2.6
Sex	Male	273	91.3	88.3 – 94.3
	Female	26	8.7	5.7 – 11.7
Education level	Informal	124	41.5	36.0 – 47.0
	Primary	142	47.5	41.9 – 53.1
	secondary	23	7.7	4.7 – 10.8
	Tertiary	10	3.3	1.3 – 5.3
Livestock keeping experience (Years)*	<2	3	1	0.0 – 2.1
	2 - 3	59	19.7	15.2 – 24.1
	>4	237	79.3	74.9 – 83.6

*Kalam, et al. (2021); CI, Confidence Interval.

This table presents the distribution of respondents by ward, age, sex, education level, and livestock-keeping experience. Most respondents were male (91.3%), within the 36–55 age range (66.6%), had primary education (47.5%), and over 4 years of livestock-keeping experience (79.3%).

### Cattle production and management of diseases

**Figure 2:**
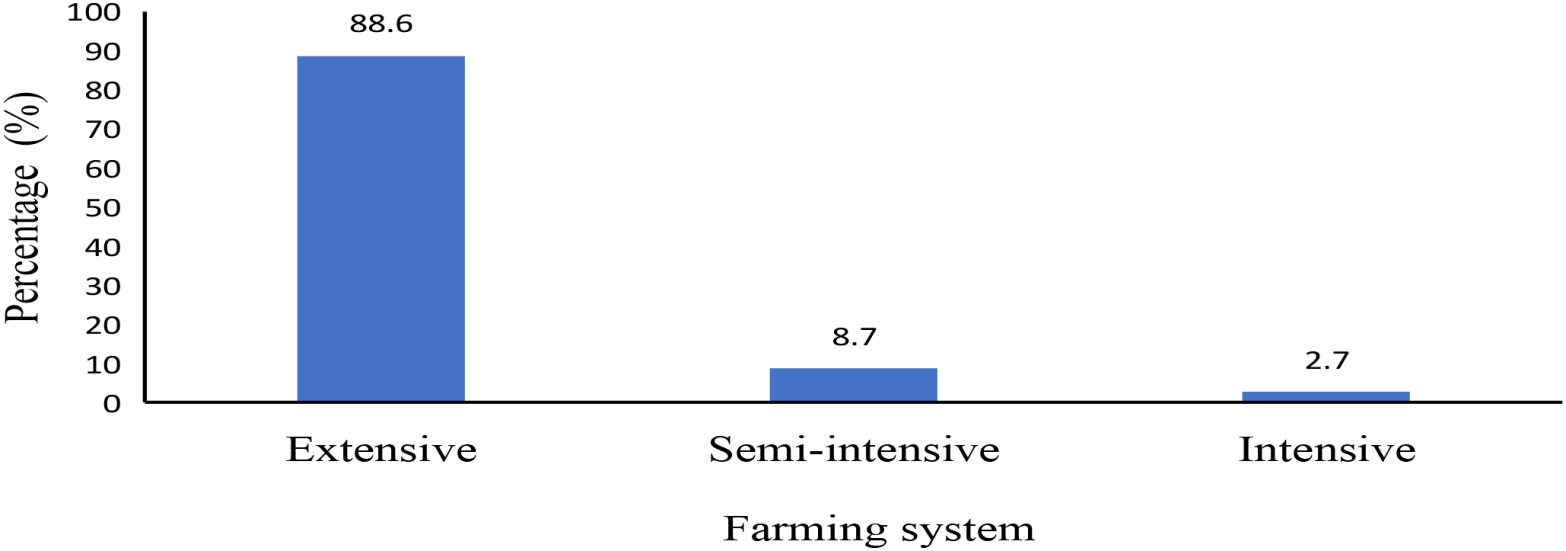
Farming systems reported by farmers.

The majority of cattle keepers practiced extensive systems (88.6%), with semi-intensive and intensive systems being practiced by 8.7 and 2.7% respectively as shown in (**Figure 2**).

**Figure 3:**
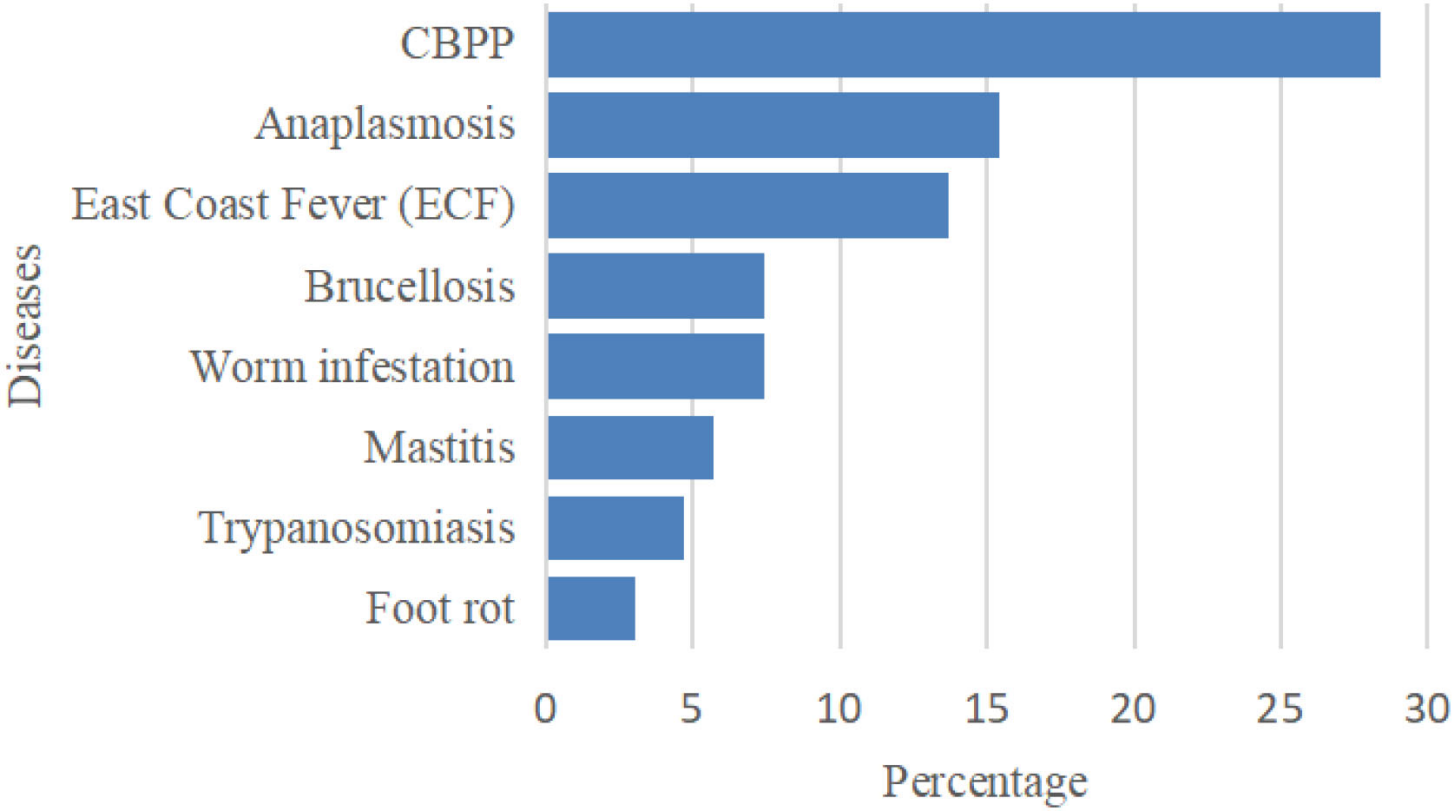
Prevalence of commonly reported cattle diseases among respondents.

The herd sizes ranged from 2 to 77 animals, with a median herd size being 28 animals. The most frequently reported cattle disease was Contagious Bovine Pleuropneumonia (28.4%), followed by Anaplasmosis (15.4%) and East Coast Fever (13.7%). Other reported conditions are shown in **Figure 3**.

**Figure 4:**
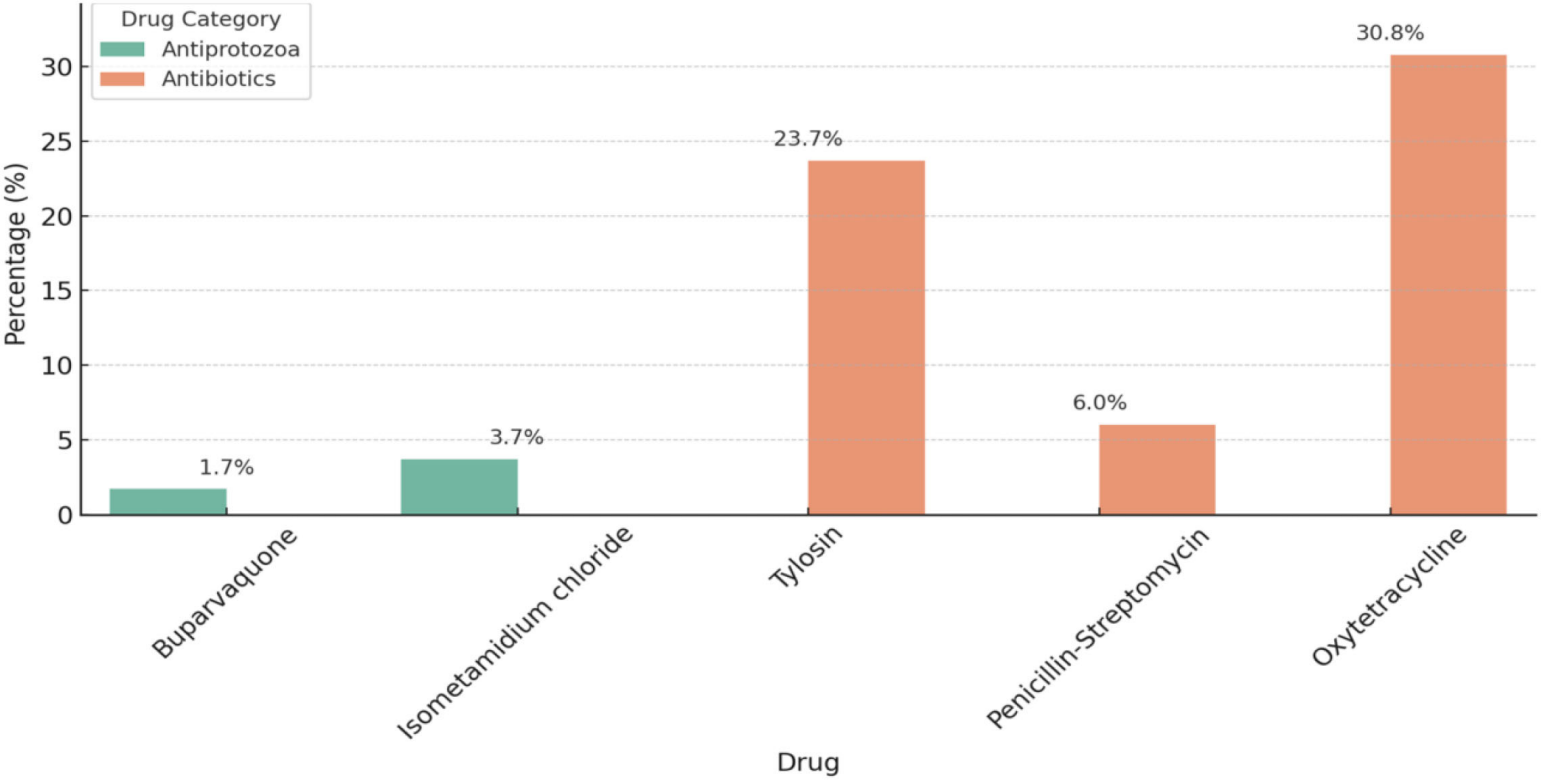
Common antimicrobials used by Mvomero cattle farmers to manage protozoal and bacterial infections.

Regarding antimicrobial use, the most reported antimicrobials used by cattle keepers included oxytetracycline (30.8%), tylosin (23.7%), Isometamedium chloride (3.7%), penicillin-streptomycin (6.0%), and buparvaquone (1.7%), as presented in **Figure 4**. Buparvaquone was used to treat East Coast Fever, Isometamedium chloride for Trypanosomiasis, Tylosin for Contagious Bovine Pleuropneumonia (CBPP), penicillin-streptomycin for Foot rot and oxytetracycline was used in treatment of each disease mentioned above possibly due to it`s broad spectrum activity.

Furthermore, our results of the disease burden by livestock production systems indicated that the extensive livestock production system accounted for the highest proportion of reported disease cases, with 265 cases (88.6%), compared to 26 cases (8.7%) in semi-intensive systems and only 8 cases (2.7%) in intensive systems. The observed trends suggest that cattle raised under extensive production systems are significantly more susceptible to diseases, likely due to greater exposure to environmental hazards, limited control over management practices, and increased interaction with other wild animals and potential disease vectors. These conditions collectively contribute to a higher disease burden in extensive systems relative to semi-intensive and intensive production models.

### Awareness of antimicrobial use and resistance

The results of 299 cattle keepers’ awareness regarding antimicrobial use, antimicrobial residues, and antimicrobial resistance (AMR) are shown in **Table 2**. The Mean awareness score was 94 (31.4%, 95%CI: 26.2-36.6). The results indicate that although a large proportion of cattle keepers 279 (93.3%,95%CI:90.8-95.9) reported knowing what antimicrobials are, their awareness on specific and technical aspects related to AMR was considerably lower with only 32 (10.7%,95%CI:7.2-14.2) of cattle keepers reporting being familiar with the concept of antimicrobial resistance, and a slightly higher proportion 43 (14.4%,95%CI:10.5-18.2) were aware that the use of antimicrobials in dairy cattle could result in antimicrobial residues in milk. Awareness of the implications of excessive antimicrobial use was also low, with only 75 (25.1%, 95%CI: 20.2-30.0) recognizing its link to drug resistance. Moreover, only 41 (13.7%, 95%CI: 9.9-17.6) of the participants were aware of the preventive measures for antimicrobial residues in milk. Overall, the findings suggest that despite high general awareness of the term “*antimicrobials*,” in-depth knowledge of residue and AMR prevention is lacking among the majority of cattle keepers.

**Table 2:** Awareness of antimicrobial use, residues, and resistance among cattle keepers in Mvomero District.

Variable	Parameter	Score	%	95% CI
Awareness	Aware of the term antimicrobials	279	93.3	90.8 – 95.9
	Aware of the term antimicrobial resistance	32	10.7	7.2 – 14.2
	Aware that the use of antimicrobials in dairy cattle can lead to antimicrobial residues in milk	43	14.4	10.5 – 18.2
	Aware that excessive use of antimicrobial drugs leads to drug resistance (AMR)	75	25.1	20.2 – 30.0
	Aware of ways used to prevent antimicrobial residue in milk	41	13.7	9.9 – 17.6
	Mean Awareness Score	94	31.4	26.2 – 36.6

CI, Confidence Interval; percentages based on N = 299.

The table summarizes awareness of antimicrobials, antimicrobial resistance (AMR), and residues in milk. While 93.3% knew what antimicrobials are, only 10.7% understood AMR, 14.4% were aware of residues in milk, and the mean awareness score was low (31.4%).

Factors associated with awareness were assessed using the multivariate linear regression model shown in **Table 3**. The model allowed for the inclusion of predictors that explained 33% of the variance in the attitude score (R² = 0.33, p < 0.001).

**Table 3:** Linear regression analysis showing socio-demographic factors associated with awareness scores.

Variable	Parameter	Coefficient (β)	Std. Error	t-test	p-value
Age	18-35	-1.48	0.49	8.87	0.003
	36-45	-1.34	0.48	7.54	0.006
	46-55	-1.22	0.48	6.29	0.013
	56-64	-1.4	0.5	7.93	0.005
	≥65 (Reference)				
Marriage	Married	-0.05	0.36	0.02	0.895
	Single	0.61	0.4	2.28	0.132
	Widow/widower	-0.43	0.48	0.8	0.372
	Divorced (Reference)				
Education	Primary	-0.08	0.15	0.31	0.581
	Secondary	0.878	0.27	9.97	0.002
	Tertiary	1.469	0.31	22.34	<0.001
	Informal (Reference)				
Experience (Years)	> 2	-1.35	0.27	71	<0.001
	2 - 3	-0.39	0.21	3.53	0.061
	>4(Reference)				
	R² = 0.33				
	Model t-statistic = 20.06, p < 0.001				

The table shows linear regression analysis of socio-demographic factors associated with awareness scores among cattle keepers. Age, higher education, and longer livestock experience were positively associated with awareness (R² = 0.33, F = 20.06, p < 0.001), while marital status showed no significant effect.

The results of multivariate linear regression analysis (**Table 3**) showed that the linear regression analysis of socio-demographic factors associated with awareness scores revealed that education, age, and livestock keeping experience were significant predictors. Farmers with tertiary education had the highest awareness scores (β = 1.469, p < 0.001), followed by those with secondary education (β = 0.878, p = 0.002), compared to those with informal education. In contrast, younger farmers exhibited lower awareness, with those aged 18–35 (β = –1.48, p = 0.003), 36–45 (β = –1.34, p = 0.006), 46–55 (β = –1.22, p = 0.013), and 56–64 (β = –1.40, p = 0.005) having significantly lower scores compared to farmers aged 65 and above. Similarly, less experienced farmers (<2 years: β = –1.35, p < 0.001; 2–3 years: β = –0.39, p = 0.061) had lower awareness than those with more than four years of livestock keeping experience. Primary education and marital status were not significantly associated with awareness. Linear regression analysis further examined the relationship between awareness and attitudes toward antimicrobial use (**Table 4**). The results showed that greater awareness was strongly associated with more positive attitudes. Specifically, awareness that antimicrobial use can lead to residues was the strongest predictor of positive attitudes (β = 6.427, p < 0.001), while awareness of antimicrobial resistance (AMR) also significantly contributed to positive attitudes (β = 2.473, p < 0.001).

**Table 4:** Linear regression analysis showing the association of awareness and attitudes.

	Variable	Coefficient (β)	Std. Error	t-test	p-value
Awareness of antimicrobial use leads to residues	Awareness of antimicrobial use leads to residues	6.427	0.369	303	<0.001
Aware AMR	Aware AMR	2.473	0.397	38.7	<0.001

The table shows the association between awareness of antimicrobial use and attitudes toward responsible use. Awareness of residues and AMR were strongly associated with positive attitudes, with regression coefficients of 6.43 and 2.47, respectively (both p < 0.001).

### Attitudes toward responsible antimicrobial use

Cattle keepers’ attitudes toward responsible antimicrobial use are presented in **Table 5**. The overall mean attitude score was 2.06 (41.2%), indicating generally low positive attitudes. The highest mean score was observed for the item “Important to follow correct dosage guidelines” (2.60; 52%), whereas the lowest scores were recorded for “Regulations on antimicrobial use are necessary” (1.88; 37.6%) and “Animals treated with antibiotics should observe waiting period” (1.89; 37.8%). Other items, including recognition of the importance of responsible antimicrobial use (1.95; 39%), awareness that training improves animal health (1.96; 39.2%), and understanding that excessive use leads to residues/AMR (2.00; 40%), also reflected low to moderate positive attitudes.

**Table 5:** Cattle keepers’ attitudes toward responsible antimicrobial use.

Attitude parameters	Mean Score	% score
Responsible antimicrobial use is important	1.95	39
Use of antimicrobials has reduced animal deaths	2.27	45.4
Important to follow the correct dosage guidelines	2.6	52
Adhere to the recommended withdrawal periods before selling milk	2.06	41.2
Excessive use leads to residues/AMR	2	40
Residues in milk pose health risks	1.93	38.6
Animals treated with antibiotics should observe a waiting period	1.89	37.8
Regulations on antimicrobial use are necessary	1.88	37.6
Training on antimicrobial stewardship improves animal health	1.96	39.2
	2.06	41.2

The table shows cattle keepers’ attitudes toward responsible antimicrobial use. Mean attitude scores ranged from 1.88 to 2.6 (37.6–52%), indicating moderate recognition of the importance of correct dosing, withdrawal periods, and training for stewardship. The overall mean attitude score was 2.06 (41.2%).

Linear regression analysis was conducted to assess socio-demographic factors associated with positive attitudes toward responsible antimicrobial use among livestock farmers (**Table 6**). The model allowed for the inclusion of predictors that explained 47% of the variance in the attitude score (R² = 0.47, p < 0.001). Male farmers had slightly higher attitude scores compared to females (β = 0.191, p = 0.0008). Attitude scores increased significantly with age, with farmers aged 36-45 (β = 0.17, p = 0.0004), 46-55 (β = 0.276, p < 0.001), 56-64 (β = 0.341, p < 0.001), and 65 and above (β = 0.396, p = 0.006) demonstrating more positive attitudes than those aged 18-35. Education was strongly associated with attitudes: farmers with secondary (β = 0.438, p < 0.001) and tertiary education (β = 1.084, p < 0.001) had substantially higher scores compared to those with informal education, whereas primary education showed a borderline effect (β = 0.065, p = 0.055). Additionally, farmers with four or more years of livestock-keeping experience exhibited higher attitude scores than those with less experience (β = 0.19, p < 0.001).

**Table 6:** Linear regression analysis showing socio-demographic factors associated with positive attitudes for responsible AMU.

Variable	Variable	Coefficient	Std Error	t-test	P-Value
Sex	Male	0.191	0.056	11.4735	0.0008
	Female*				
Age	36-45	0.17	0.047	12.9456	0.0004
	46-55	0.276	0.047	34.5216	P<0.001
	56-64	0.341	0.056	36.7273	P<0.001
	65 and above	0.396	0.143	7.6394	0.006
	18-35*				
Education level	Primary	0.065	0.034	3.7074	0.055
	Secondary	0.438	0.062	49.0952	P<0.001
	Tertiary	1.084	0.09	144.3423	P<0.001
	Informal*				
Livestock keeping experience	≥4	0.19	0.04	22.8754	0.000003
	≤4*				
	CONSTANT	1.442	0.078	343.6101	P<0.001

*Reference category, R² = 0.47.

The table shows linear regression analysis of factors associated with positive attitudes toward responsible antimicrobial use. Male sex, older age, higher education, and longer livestock-keeping experience were positively associated with attitude scores (all p < 0.001), while younger and less educated respondents showed lower scores.

### Practices toward responsible antimicrobial use

The results in **Table 7** indicate that the majority (99.7%, 95%CI: 99.1-100) of cattle keepers used antimicrobials in their animals for management of diseases, which is a potential source of antimicrobial residues. Also 77.6% (95%CI: 72.8-82.3) applied manure to farm fields as organic fertilizer, while 22.4% (95%CI: 17.7-27.2) sold it.

**Table 7:** Practices on antimicrobial usage and management among cattle farmers in Mvomero district.

Parameter	Aspect	Frequency(n)	%	95% CI
	Use antimicrobials	298	99.7	99.1 – 100
Antimicrobial use and disposal	Dispose of expired antibiotics	7	2.3	0.6 – 4.0
	Administers drugs personally	72	24.1	19.1 – 29.0
	Extended ECF dosage administration	15	36.6	21.8 – 51.3
	Manure disposal-fertilizer	232	77.6	72.8 – 82.3
	Manure disposal-selling	67	22.4	17.7 – 27.2
Overall hygienic conditions	Hand wash before touching the animal	174	58.2	52.5 – 63.8
	Unsatisfactory staple washing	174	58.2	52.5 – 63.8
	Use of Soap when washing hands	16	5.4	2.8 – 8.0
	Use of Footbath	17	5.7	3.1 – 8.3
Medical records	Respondents who keep medication records	63	21.1	16.4 – 25.8
	Type of antimicrobial used	44	69.8	57.1 – 82.5
	Dosage	28	44.4	30.0 – 58.7
	Duration of treatment	21	33.3	19.8 – 46.9
	Reason of use	38	60.3	46.2 – 74.4

CI, Confidence Interval; percentages based on N = 299.

This table describes antimicrobial use, disposal, hygiene, and record-keeping practices. Almost all respondents reported using antimicrobials (99.7%), but only 2.3% disposed of expired antibiotics correctly. Hygiene practices were suboptimal, with only 5.4% using soap for handwashing. Medication record-keeping was low (21.1%).

In terms of hygiene, 58.2%(95%CI:52.5-63.8) of cattle keepers practiced hand washing before milking, but only 5.4%(95%CI:2.8-8.0) used soap and 5.7%(95%CI:3.1-8.3) used footbaths, indicating generally poor hygiene practices.

Only 21.1%(95%CI=16.4-25.8) of cattle keepers kept records of antimicrobial use, and even fewer documented essential details such as dosage, treatment duration, or reasons for use.

Despite widespread antimicrobial use and some hygiene awareness, overall practices were inadequate and fell short of standards for responsible antimicrobial management.

### Preventive strategies and misconceptions regarding antimicrobial residues

**Figure 5:**
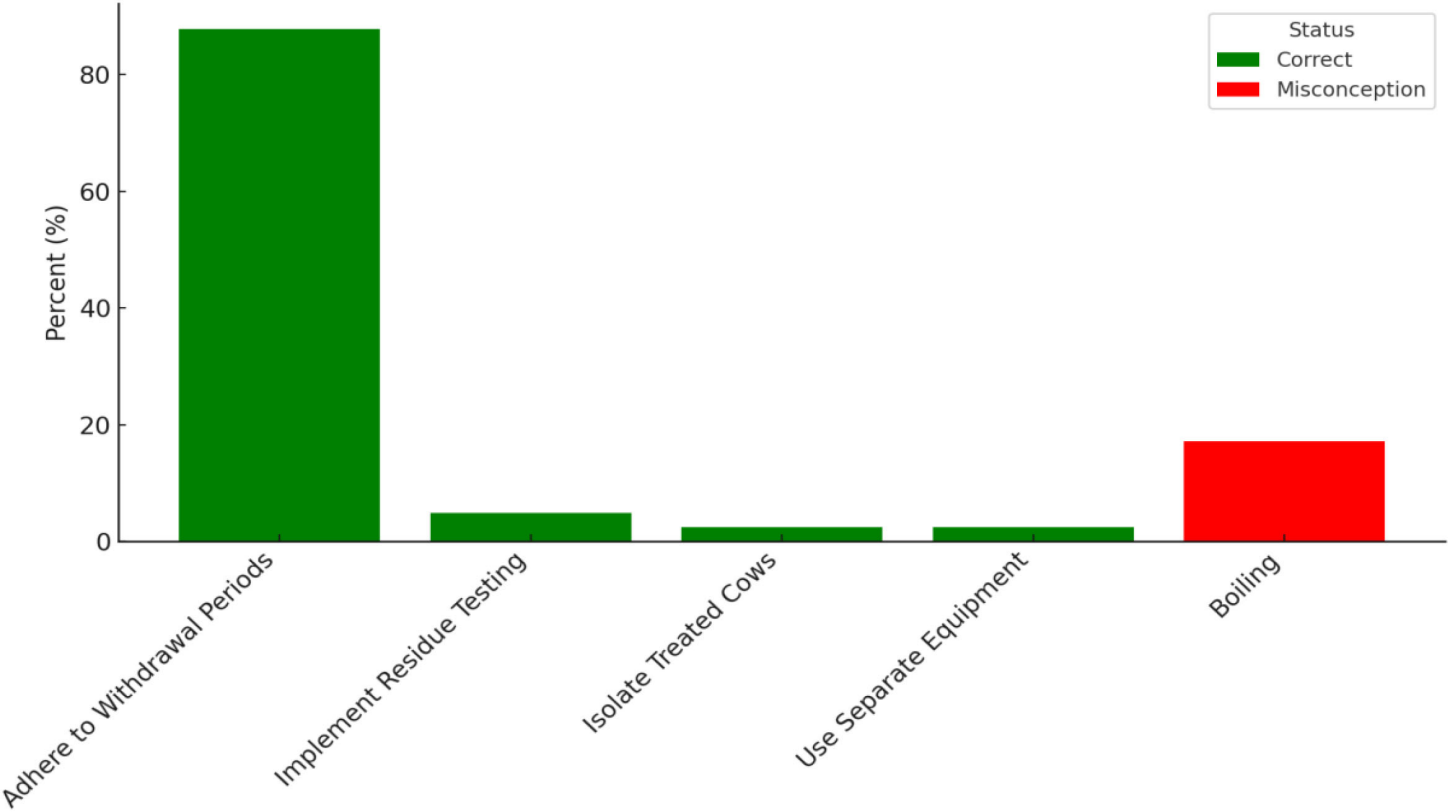
Preventive strategies and misconceptions regarding antimicrobial residues.

Among the 41 cattle keepers who were aware of preventive strategies for antimicrobial residues, adherence to withdrawal periods was the most frequently cited correct measure (87.8%) (**Figure 5**). Other appropriate practices mentioned included isolating treated cows (2.4%) and using separate milking equipment (2.4%). However, 17.1% of cattle keepers believed that boiling milk could eliminate antimicrobial residues, indicating a prevalent misconception and highlighting a gap in awareness regarding proper residue prevention methods.

### Key informants’ results among animal health practitioners and drug retailers

Key informants identified several major issues affecting antimicrobial use among livestock practitioners and veterinary drug sellers (**Table 8**). Non-prescription sales of antimicrobials were reported by all drug sellers (13/13) and most practitioners (9/10), indicating frequent access without professional oversight. Reliance on clinical signs rather than laboratory diagnostics was reported by all practitioners (10/10) and by some drug sellers (3/10), which suggest frequent empirical treatment. Weak regulatory enforcement, combined with client or farmer pressure, was reported by the majority of practitioners (8/10) and drug sellers (11/13), which may contribute to inappropriate antimicrobial dispensing. Other challenges included limited knowledge of antimicrobial resistance and stewardship, as well as inconsistent record-keeping, which may compromise treatment monitoring and traceability. These findings indicate that non-prescription access, empirical treatment practices, and regulatory gaps are key drivers of antimicrobial misuse in the livestock sector. The Table matrix linking quantitative survey data with the qualitative thematic analysis, showing frequencies and highlighting major issues, is shown in **Appendix 1**.

**Table 8:** Findings from key informant interviews on antimicrobial use practices and challenges.

Theme	Description	Animal health practitioners (n=10)	Drug sellers (n=13)
Diagnostic Practices	Reliance on clinical signs; minimal use of laboratory diagnostics	10/10	3/10
Record-Keeping & Documentation	Poor treatment logs, drug inventories, and adverse event reporting	7/10	4/13
Knowledge & Awareness of AMR & Stewardship	Limited understanding of antimicrobial resistance, residues, and withdrawal periods	6/10	3/13
Professional Oversight & Training	Lack of formal veterinary training; minimal supervision	2/10	1/13
Regulatory Gaps & Client Pressure	Weak enforcement of guidelines; farmer/client pressure to dispense antibiotics	8/10	11/13
Access & Distribution of Antimicrobials	Widespread non-prescription sales of antimicrobials	9/10	13/13

The table shows key findings from interviews with animal health practitioners (n = 10) and drug sellers (n = 13) on antimicrobial use practices and challenges. Most practitioners relied on clinical signs (10/10), and non-prescription sales were common among drug sellers (13/13). Limited knowledge of AMR, poor record-keeping, regulatory gaps, and client pressure were frequently reported.

## Discussion

This study examined factors influencing antimicrobial use, awareness, attitudes and practices among cattle keepers in Mvomero District, as well as AMS practices and challenges reported by animal health practitioners and veterinary drug retailers. Overall, awareness of AMR and residues was low, adherence to recommended use guidelines was limited, and empirical treatment practices were common (Azabo et al., 2022). Education, age, and livestock-keeping experience shaped awareness and attitudes, while stewardship challenges included limited diagnostic capacity, non-prescription sales, weak regulatory enforcement, and client-driven pressure, all contributing to inappropriate antimicrobial use and the risk of residues in animal products (Mangesho et al., 2021; Jaime et al., 2022).

Most cattle keepers reported general awareness of antimicrobials, but technical knowledge of AMR, residues, and preventive measures was limited. This indicates that knowledge is often superficial, focusing on recognition rather than correct use and associated risks. Similar patterns have been reported in Tanzania, where many poultry and cattle farmers lacked understanding of dosing and withdrawal periods (Azabo et al., 2022) (Frumence et al., 2021). Found that many veterinary paraprofessionals had not received formal training on antimicrobial use and resistance, limiting their ability to advise farmers. Comparable gaps in technical knowledge have also been observed in Rwanda and Kenya, where familiarity with antimicrobials does not always translate into informed stewardship (Frumence et al., 2021; Kibooga et al., 2023).

Socio-demographic factors influenced awareness and attitudes. Older farmers and those with higher education and more livestock experience had more responsible attitudes. Older cattle keepers may have accumulated experience managing livestock challenges and interacting with veterinary services, which shapes their understanding of antimicrobial use and resistance (Nonga et al., 2010; Kimera et al., 2020). Younger farmers often rely on peers or economic priorities, which may lead to practices such as self-prescription or incomplete dosing (Jaime et al., 2022). Education improves access to information, understanding of technical concepts, and decision-making, which supports rational antimicrobial use (Tang et al., 2023). Studies in Uganda and Tanzania show that higher education levels are linked with better knowledge and attitudes toward antimicrobial stewardship (Kibooga et al., 2023; Mapunjo et al., 2025).

Experience in livestock management also shaped attitudes. Farmers with longer experience showed more careful practices, likely due to repeated encounters with treatment failures, costs of disease, and advice from animal health practitioners. Experience acts as an informal learning mechanism, reinforcing awareness of antimicrobial risks (Kemp et al., 2021; Hirwa et al., 2024). Gender influenced attitudes, with male farmers showing slightly more positive attitudes, possibly due to gender roles in herd management and antimicrobial procurement, which affects exposure to guidance and training (Collineau et al., 2017).

Empirical treatment and limited diagnostic capacity among practitioners and drug sellers were common. Reliance on clinical signs without laboratory confirmation can promote inappropriate antimicrobial use, increasing the risk of residues and resistance (Katakweba et al., 2018). Regulatory gaps, client pressure, and non-prescription sales further contribute to misuse (Loosli et al., 2021). Poor record-keeping and low adherence to withdrawal periods heightens the risk of antimicrobial residues in animal products (Ngumbi and Silayo, 2017).

Comparable patterns have been observed across sub-Saharan Africa. In Zanzibar, poultry sellers had low technical knowledge of AMR yet maintained relatively positive attitudes (Nkinda et al., 2022). In Zambia, awareness was strongly influenced by education and experience. Systematic reviews indicate that low technical awareness contributes to improper antimicrobial use and higher AMR burden across African livestock systems (Nthambi et al., 2023). These findings highlight the need for interventions that promote behavior change, improve access to veterinary guidance and consider socio-demographic factors.

Antimicrobial stewardship (AMS) in Mvomero faces challenges from limited diagnostics, empirical treatment, weak regulation, poor record-keeping, and client pressure. These issues are common in low- and middle-income countries, contributing to misuse and resistance (Zeru et al., 2019). Effective AMS requires combining education, governance, and diagnostic support rather than relying solely on awareness campaigns (OIE, 2020; USAID MTaPS Tanzania, 2022).

This study reinforces the need for a One Health framework to tackle AMR in rural Tanzania. Interconnections between human health, animal health, and environmental contamination were evident in drug disposal, manure use, and hygiene practices. Findings support calls for integrated surveillance and stronger regulatory enforcement.

To mitigate AMR risks, Tanzania’s NAP-AMR must be fully operationalized with increased investment in veterinary infrastructure, enforcement of prescription-only policies including antimicrobial categorization, and culturally relevant health education [health and fisheries]. Strengthening local government roles in veterinary public health governance is vital to embed stewardship principles into livestock management (WHO, W. H. O. et al., 2019).

## Recommendations

To improve antimicrobial stewardship, cattle keepers should be educated on the risks of residues and resistance and encouraged to follow recommended dosing and withdrawal periods. Veterinary oversight should be strengthened, and drug sellers and practitioners encouraged to base treatments on diagnostics. Regulatory enforcement should be improved to reduce non-prescription access and manage client pressure. Promoting proper record-keeping, hygiene, and manure management can also help reduce antimicrobial residues and resistance.

## Study limitation

The self-reported data on knowledge, attitudes, and practices may be subjected to recall or social desirability bias. Also, most key informants, particularly veterinary drug retailers and animal health practitioners, were reluctant to be audio-recorded during interviews. This limited the depth and richness of qualitative data, as interviewers had to rely solely on note-taking, which may have missed some nuances in responses.

## Conclusion

This study highlights gaps in knowledge, attitudes, and practices on antimicrobial use among cattle keepers in Mvomero District. Most farmers recognize antimicrobials, but few understand AMR, residues, or preventive measures. Education, age, and livestock-keeping experience influenced awareness and attitudes. Interviews with animal health practitioners and drug sellers revealed that empirical treatment, limited diagnostics, weak regulations, client pressure, and non-prescription sales hinder responsible use. Therefore; interventions are needed to provide farmer training, strengthen veterinary guidance, enforce regulations, and monitor drug use to reduce drug residue and AMR risk to protect animal and public health.

## Data Availability

The original contributions presented in the study are included in the article/supplementary material. Further inquiries can be directed to the corresponding author.

## Appendix 1

**Table A1:** Table matrix linking quantitative survey data with the qualitative thematic analysis, showing frequencies and highlighting major issues.

Theme/Variable	Quantitative evidence	Qualitative evidence	Frequency (KI: AHP/VDR)	Interpretation
Diagnostic Practices	Low adherence to evidence-based diagnosis; treatment is often empirical	Reliance on clinical signs; minimal use of lab diagnostics	10/10 AHP, 3/13 VDR	Empirical treatment may lead to misuse of antimicrobials
Record-Keeping & Documentation	Only 21.1% of cattle keepers kept any records; dosage logs 44.4%, treatment duration 33.3%	Poor treatment logs, drug inventories, adverse event reporting	7/10 AHP, 4/13 VDR	Weak traceability and pharmacovigilance
Knowledge & Awareness of AMR & Stewardship	Awareness scores positively associated with higher education (Secondary: β=0.878, p=0.002; Tertiary: β=1.469, p<0.001); overall attitude mean 2.06/5	Limited understanding of AMR, residues, withdrawal periods	6/10 AHP, 3/13 VDR	Knowledge gaps influence attitudes and practices
Professional Oversight & Training	Survey indicated poor adherence to recommended practices	Lack of formal veterinary training; minimal supervision	2/10 AHP, 1/13 VDR	Limited oversight contributes to non-compliant AMU
Regulatory Gaps & Client Pressure	High proportion of self-medication, extended treatment durations, poor withdrawal adherence	Weak enforcement of guidelines; farmer/client pressure to dispense antibiotics	8/10 AHP, 11/13 VDR	Structural and regulatory barriers exacerbate misuse
Access & Distribution of Antimicrobials	99.7% of cattle keepers used antimicrobials; 78.9% had no prescription records	Non-prescription sales common; unrestricted access	9/10 AHP, 13/13 VDR	Easy access drives inappropriate use and AMR risk
Hygiene & Biosecurity Practices	Handwashing 5.4%, footbaths 5.7%, withdrawal period adherence low	Reported improper handling and disposal of drugs, manure used as fertilizer	N/A	Environmental contamination and residue accumulation risk

## References

[B1] Akhila P. Christabel S. Alva J. (2025). Antibiotics awareness: exploring the knowledge and attitude towards the usage and resistance among non-health professional students. Crit. Public Health 35, 1–8. doi: 10.1080/09581596.2025.2500114

[B2] Azabo R. Mshana S. Matee M. Kimera S. I. (2022). Antimicrobial usage in cattle and poultry production in Dar es Salaam, Tanzania: pattern and quantity. BMC Vet. Res. 18, 1–12. doi: 10.1186/s12917-021-03056-9, PMID: 34980101 PMC8722348

[B3] Caudell M. Mangesho P. E. Mwakapeje E. R. Dorado-García A. Kabali E. Price C. . (2022). Narratives of veterinary drug use in northern Tanzania and consequences for drug stewardship strategies in low-income and middle-income countries. BMJ Global Health 7, 1–12. doi: 10.1136/bmjgh-2021-006958, PMID: 35058305 PMC8772431

[B4] Collineau L. Belloc C. Stärk K. D. C. Hémonic A. Postma M. Dewulf J. . (2017). Guidance on the selection of appropriate indicators for quantification of antimicrobial usage in humans and animals. Zoonoses Public Health 64, 165–184. doi: 10.1111/zph.12298, PMID: 27592024

[B5] Coque T. M. Cantón R. Pérez-Cobas A. E. Fernández-de-Bobadilla M. D. Baquero F. (2023). Antimicrobial resistance in the global health network: known unknowns and challenges for efficient responses in the 21st century. Microorganisms 11, 1–32. doi: 10.3390/microorganisms11041050, PMID: 37110473 PMC10144039

[B6] Food and Agriculture Organization of the Nations (FAO) World Health Organization (OMS) (2018). Evaluation of certain veterinary drug residues in food: Eighty-fifth report of the Joint FAO/WHO Expert Committee on Food Additives. WHO technical report series; no. 1008. Available online at: https://apps.who.int/iris/bitstream/handle/10665/259895/9789241210171-eng.pdf (Accessed April 22, 2025)., PMID:

[B7] Frida M. Resto M. Faith M. Kennedy C. (2017). Oxytetracycline residue levels in beef in Dodoma region, Tanzania. Afr. J. Food Sci. 11, 40–43. doi: 10.5897/ajfs2016.1532

[B8] Frumence G. Mboera L. E. G. Sindato C. Katale B. Z. Kimera S. Metta E. . (2021). The governance and implementation of the national action plan on antimicrobial resistance in Tanzania: A qualitative study. Antibiotics. 10, 1–16. doi: 10.3390/antibiotics10030273, PMID: 33803077 PMC7998560

[B9] Gilbert W. Thomas L. F. Coyne L. Rushton J. (2021). Review: Mitigating the risks posed by intensification in livestock production: the examples of antimicrobial resistance and zoonoses. Animal 15, 100123. doi: 10.1016/j.animal.2020.100123, PMID: 33573940

[B10] Health, M. of;, & Livestock and Fisheries, M. of (2022). The National Action Plan on Antimicrobial Resistance 2023 -2028 Vol. 136 (Tanzania: Ministry of Health Health).

[B11] Hirwa E. M. Mujawamariya G. Shimelash N. Shyaka A. (2024). Evaluation of cattle farmers’ knowledge, attitudes, and practices regarding antimicrobial use and antimicrobial resistance in Rwanda. PloS One 19, 1–22. doi: 10.1371/journal.pone.0300742, PMID: 38603685 PMC11008905

[B12] Jaime G. Hobeika A. Figuié M. (2022). Access to veterinary drugs in sub-Saharan Africa: roadblocks and current solutions. Front. Vet. Sci. 8. doi: 10.3389/fvets.2021.558973, PMID: 35356415 PMC8959935

[B13] Katakweba A. A. S. Muhairwa A. P. Lupindu A. M. Damborg P. Rosenkrantz J. T. Minga U. M. . (2018). First report on a randomized investigation of antimicrobial resistance in fecal indicator bacteria from livestock, poultry, and humans in Tanzania. Microb. Drug Resist. 24, 260–268. doi: 10.1089/mdr.2016.0297, PMID: 28759321

[B14] Kemp S. A. Pinchbeck G. L. Fèvre E. M. Williams N. J. (2021). Cross-sectional survey of the knowledge, attitudes, and practices of antimicrobial users and providers in an area of high-density livestock-human population in western Kenya. Front. Vet. Sci. 8. doi: 10.3389/fvets.2021.727365, PMID: 34621809 PMC8490823

[B15] Kibooga C. Asiimwe R. Nakiyemba C. (2023). Use of antibiotics in livestock production in Uganda. International fund for Agriculture Development. 7, 1–7.

[B16] Kimera Z. I. Mshana S. E. Rweyemamu M. M. Mboera L. E. G. Matee M. I. N. (2020). Antimicrobial use and resistance in food-producing animals and the environment: An African perspective. Antimicrob. Resist. Infect. Control 9, 1–12. doi: 10.1186/s13756-020-0697-x, PMID: 32122406 PMC7053060

[B17] Kisoo L. Muloi D. M. Oguta W. Ronoh D. Kirwa L. Akoko J. . (2023). Practices and drivers for antibiotic use in cattle production systems in Kenya. One Health 17, 1–10. doi: 10.1016/j.onehlt.2023.100646, PMID: 38024269 PMC10665206

[B18] Loosli K. Davis A. Muwonge A. Lembo T. (2021). Addressing antimicrobial resistance by improving access and quality of care—a review of the literature from east africa. PloS Negl. Trop. Dis. 15, 1–17. doi: 10.1371/journal.pntd.0009529, PMID: 34292932 PMC8297743

[B19] Mangesho P. E. Caudell M. A. Mwakapeje E. R. Ole-Neselle M. Kabali E. Obonyo M. . (2021). We are doctors”: Drivers of animal health practices among Maasai pastoralists and implications for antimicrobial use and antimicrobial resistance. Prev. Vet. Med. 188, 105266. doi: 10.1016/j.prevetmed.2021.105266, PMID: 33517159

[B20] Mapunjo S. Mbwasi R. Nkiligi E. A. Wilbroad A. Francis E. N. Msovela K. . (2025). National consumption of antimicrobials in Tanzania: 2020-2022. JAC Antimicrob. Resist. 7, 1–11. doi: 10.1093/jacamr/dlaf026, PMID: 40046067 PMC11879584

[B21] Mdegela R. H. Mwakapeje E. R. Rubegwa B. Gebeyehu D. T. Niyigena S. Msambichaka V. . (2021). Antimicrobial use, residues, resistance and governance in the food and agriculture sectors, Tanzania. Antibiotics 10, 1–23. doi: 10.3390/antibiotics10040454, PMID: 33923689 PMC8073917

[B22] Mpina M. C. Mgonja F. R. (2024). Assessment of stakeholders’ knowledge, attitude, and practice in cattle production and supply chain on antimicrobial usage in Kilosa District, Morogoro. East Afr. J. Sci. Technol. Innovation 5, 1–17. doi: 10.37425/6815c216

[B23] Ngumbi A. F. Silayo R. S. (2017). A cross-sectional study on the use and misuse of trypanocides in selected pastoral and agropastoral areas of eastern and northeastern Tanzania. Parasites Vectors 10, 1–9. doi: 10.1186/s13071-017-2544-3, PMID: 29246241 PMC5731095

[B24] Nkinda L. Mwakawanga D. L. Kibwana U. O. Mikomangwa W. P. Myemba D. T. Sirili N. . (2022). Implementation of antibiotic stewardship programmes in paediatric patients in regional referral hospitals in Tanzania: experience from prescribers and dispensers. JAC Antimicrob. Resist. 4, 1–8. doi: 10.1093/jacamr/dlac118, PMID: 36439992 PMC9683393

[B25] Nonga H. E. Simon C. Karimuribo E. D. Mdegela R. H. (2010). Assessment of antimicrobial usage and residues in commercial chicken eggs from smallholder poultry keepers in morogoro municipality, Tanzania. Zoonosis pubic health 4, 339–344. doi: 10.1111/j.1863-2378.2008.01226.x, PMID: 19486498

[B26] Nthambi M. Lembo T. Davis A. Nasuwa F. Mmbaga B. T. Matthews L. . (2023). Quantifying farmers’ preferences for antimicrobial use for livestock diseases in northern Tanzania. Q Open 3, 1–28. doi: 10.1093/qopen/qoac032

[B27] OIE (2020). “ OIE Standards, Guidelines and Resolutions on Antimicrobial Resistance and the use of antimicrobial agents,” in International Office of Epizootics. ( Terrestrial Animal health code) Available online at: www.oie.int (Accessed May 19, 2025).

[B28] Pinto Ferreira J. Battaglia D. Dorado García A. Tempelman K. A. Bullon C. Motriuc N. . (2022). Achieving antimicrobial stewardship on the global scale: challenges and opportunities. Microorganisms 10, 1–16. doi: 10.3390/microorganisms10081599, PMID: 36014017 PMC9412511

[B29] Tang K. W. K. Millar B. C. Moore J. E. (2023). Antimicrobial resistance (AMR). Br. J. Biomed. Sci. 80. doi: 10.3389/bjbs.2023.11387, PMID: 37448857 PMC10336207

[B30] URT (2017). Mvomero district investment profile. (Tanzania: MTaps) 1–37.

[B31] USAID MTaPS Tanzania (2022). Technical Brief: Strengthening Antimicrobial Stewardship (AMS) in Tanzania. (Tanzania: MTaps) 1–6.

[B32] WHO, W. H. O FAO, F. and A. O. of the U. N OIE, W. O. for A. H (2019). Monitoring and evaluation of the global action plan on antimicrobial resistance. Available online at: https://apps.who.int/iris/bitstream/handle/10665/325006/9789241515665-eng.pdf?sequence=1&isAllowed=y (Accessed April 22, 2025).

[B33] Zeru H. Gemechu A. Andualem T. Seid H. Mengesha F. (2019). Awareness of veterinary drug retail outlets on antimicrobial resistance and its containment strategy in Ethiopia. Pharmacovigilance Pharmacoepidemiol. 2, 9–13. doi: 10.33805/2638-8235.111

